# Intercalated Motifs Contribute to Transcription Regulation in an Acidosis Model

**DOI:** 10.3390/biom16071038

**Published:** 2026-07-16

**Authors:** Varvara Sapozhnikova, Ekaterina Knizhnik, Dmitriy Shirokov, Yuri Khodarovich, Julia Khomyakova, Margarita Bogomiakova, Alexander Tikhomirov, Andrey Shchekotikhin, Anna Varizhuk, Vladimir Tsvetkov

**Affiliations:** 1 Lopukhin Federal Research and Clinical Center of Physical-Chemical Medicine of Federal Medical Biological Agency, 119435 Moscow, Russia; sapozhnikova.v@rcpcm.ru (V.S.); knizhnik.e@rcpcm.ru (E.K.); dmitry.a.shirokov@gmail.com (D.S.); khomyakova.j@rcpcm.ru (J.K.); bogomyakova.m@rcpcm.ru (M.B.); 2 K.I. Skryabin Moscow State Academy of Veterinary Medicine and Biotechnology, 109472 Moscow, Russia; 3 Shemyakin-Ovchinnikov Institute of Bioorganic Chemistry, Russian Academy of Sciences, 117997 Moscow, Russia; khodarovich@mail.ru; 4 Research and Educational Resource Center for Cellular Technologies of the Peoples’ Friendship University of Russia, 117198 Moscow, Russia; 5 Gause Institute of New Antibiotics, 119021 Moscow, Russia; tikhomirov.chem@gmail.com (A.T.); shchekotikhin@mail.ru (A.S.); 6 A. V. Topchiev Institute of Petrochemical Synthesis RAS, 119991 Moscow, Russia

**Keywords:** intercalated motif, transcription regulation, acidosis, early response genes

## Abstract

The formation and function of intercalated motifs (iMs) in cytosine-rich regions of the human genome have long been debated. While these pH-sensitive structures are unlikely to govern gene expression in normal tissues, their contribution to acidosis-specific transcriptional reprogramming cannot be ruled out. Here, we focused on previously reported iM-forming sequences from the regulatory regions of early response genes. We tested their impact on transcription via luciferase-based assays under normal conditions, as well as under conditions favoring or disfavoring iMs (acidic media or iM-destabilizing ligands, respectively). We verified and elucidated the effects of pH and ligands by optical methods and molecular modeling, respectively. Our data indicate that iMs tend to repress reporter gene expression in response to acidification, and this effect could be reversed by selective ligands, including an anticancer drug candidate. These results highlight the potential of targeting iMs to mitigate the pathogenic response to acidosis.

## 1. Introduction

Intercalated motifs (iMs) are noncanonical secondary structures of C-rich DNA composed of parallel duplexes in an antiparallel orientation. Each duplex is held together by hydrogen bonds between hemiprotonated cytosine residues from C-tracts [1,2]. This accounts for the pH-dependence of iM formation [3]. Van der Waals interactions between deoxyribose (but not ribose) residues of adjacent C-tracts from different duplexes are thermodynamically favorable [4], which explains the enhanced stability of iMs compared to isolated parallel duplexes in DNA and the depletion of stable iMs in RNA [5]. Genomic iMs can be intermolecular (e.g., in centromeres [6]) or intramolecular (e.g., in promoters and untranslated regions, UTRs [7,8]). Intramolecular folding requires the presence of four C_3+_ tracts separated by loops of up to six nucleotide (nt) residues (the central loop can be longer [2]. Arrays of multiple C-tracts exhibit enhanced conformational polymorphism, which has been revealed by V.B. Tsvetkov [9]. If the C-tracts are relatively long (typically ≥ 5-nt) [10] and the loops are reasonably short [11] or form additional iM-core-stabilizing pairs or tetrads [12,13], the structure may sustain physiological pH. However, most iMs are only thermodynamically stable in mildly acidic media, which calls their biological relevance into question. This issue is further complicated by the fact that the determinants of iM formation in the genomic context may differ from those obtained in vitro [14]. It has been debated whether iMs create steric hindrance for guanine quadruplexes (G4s) in complementary strands, and whether these structures are mutually exclusive [15]. V.B. Tsvetkov showed the absence of steric hindrance in most cases [9]. Recent studies highlight the interdependence of G4s and iMs in the cell cycle, rather than a competition between them [16].

Methodological advances that paved the way for the current understanding of genomic iMs include bioinformatic predictions [10,17], the development of iM-specific antibodies (iMAbs) [18], the adaptation of in-cell nuclear magnetic resonance (NMR) spectroscopy for iM monitoring in nuclei [19], mapping in eukaryotic genomes using immunoprecipitation and sequencing (iM-iP-seq) [20], and the optimization of the mapping technique using cleavage under targets and tagmentation (CUT&Tag) [8]. Specifically, mapping disclosed iM association with regulatory regions of the human genome [20]; immunocytochemistry with iMAbs enabled visualization of genomic iMs in human cells and highlighted their enrichment in the G1 phase of the cell cycle [18]; and NMR confirmed that synthetic iMs are at least partially folded in the transfected cells’ nuclei [19]. However, a recent alignment of NMR-based studies with CUT&Tag-based mapping in the human genome [21] revealed that the vast majority of sites with confirmed iM-folding potential under CUT&Tag conditions (prolonged storage at 4 °C) are likely unfolded at the physiological temperature (37 °C) and native pH (7.4), even when genomic DNA is unwound (e.g., in R-loops formed during transcription). These results strongly argue against the participation of iMs in transcription regulation under normal conditions. However, they do not exclude the prevalence of iMs and their specific roles in an acidified microenvironment, such as the contribution to acidosis-driven transcriptional reprogramming associated with aggressive tumor phenotypes [22]. While transient metabolic changes or early oncogenesis with Warburg effect-driven reversion of the intracellular/extracellular pH gradient suggest no nucleoplasm acidification [23], chronic neuroinflammation or prolonged hypoxia in solid tumors can lead to severe acidosis with an intracellular pH of less than 6.6 [24,25,26]. Intracellular acidification reportedly occurs at the tumor periphery, where carbonic anhydrase IX levels are insufficient to balance the accumulation of extracellular lactate and maintain the intracellular/extracellular pH gradient [27]. While transient cytosolic pH changes within 0.1–0.2 units are localized and controlled by intracellular acid-base transporters [28], pronounced acidification eventually reaches the nucleus, as suggested by recent studies using optical pH sensors [29]. Theoretically, this creates prerequisites for iM formation and interference with transcription.

Thus far, the impact of iMs on transcription in cell models has only been verified under normal conditions for a limited number of genes [30]. In most cases, these studies did not distinguish iM-related effects from those of G4s or other noncanonical structures. Notable exceptions include the promoters of the oncogenes *KRAS* [31] and *MYC* [32], the tumor suppressor *Bcl2* [33], and the insulin-linked polymorphic repeat region ILPR [34]. iMs in the promoters of these genes tend to enhance or fine-tune transcription in a protein- or small-molecule-dependent manner (e.g., upon binding to the heterogeneous nuclear ribonucleoprotein K [31,32] or a cholestane derivative [33]). Importantly, they exist in dynamic equilibrium with hairpins [31,33] and G4s (likely shifted rather than iM-opposing) [32,34]. This equilibrium is sensitive not only to iM binders but also to dsDNA-stabilizing factors such as SP1 and G4-binders such as nucleolin [32]. Thus, iMs linked to transcription activation act indirectly as elements of more complex conformational switches. Most well-characterized iMs have been reported to downregulate transcription, but further investigation is needed. One example is the iM structure from the Acetyl-CoA Carboxylase 1 (ACC1) promoter [35]. Its repressive role in ACC1 regulation was demonstrated using luciferase assays in HeLa cells under native conditions (pH 7.4). This contradicts the loss of the ACC1 iM effect in primer extension assays at pH above 6.5. The authors attributed the superior performance of iMs in cells to molecular crowding. However, the crowding effect does not fully resolve the discrepancy with recent in-cell NMR data, which revealed iM depletion in HeLa cells under native conditions [21]. Furthermore, crowding stabilizes both iMs and G4s [36], so the predominant impact of G4s on reporter gene expression cannot be excluded. Our goal was to verify the direct impact of iMs on reporter gene expression using a combination of assays under conditions that favor or disfavor iMs. The former required an acute acidosis model, while the latter required the use of iM-disrupting and/or G4-stabilizing ligands. We aimed to complement previous assays with G4/iM-binders and focused on small molecules with excellent selectivity [37,38]. Additionally, we present the first direct tests of iMs as transcription regulators in acidosis.

## 2. Materials and Methods

### 2.1. Oligonucleotides, Ligands, Circular Dichroism (CD) Measurements, and Melting Assays

Sequences of intercalated motifs (iMs) from promoter regions or 5′-untranslated regions of oncogenesis-related genes were selected from CUT&Tag peaks [GSE220882] [8] using the G4/iM-Hunter application [39], which is available at the web server DNA Analyses tools (https://bioinformatics.ibp.cz/#/, accessed on 10 July 2026, Brno, Czech Republic). Their extended versions (iMs with 5-nt genomic flanks, 95% purity after HPLC purification) were obtained from Litekh (Moscow, Russia). The sequences are shown in Table 1. Concentrations of all oligonucleotides were verified by absorbance measurements at 260 nm using the following molar extinction values for deoxyribonucleotide residues: 15,200 M^−1^cm^−1^ (A), 8400 M^−1^cm^−1^ (T), 12,010 M^−1^cm^−1^ (G), and 7050 M^−1^cm^−1^ (C). For verification of pH-dependent iM folding, the oligonucleotides were dissolved in 10 mM sodium phosphate (pH 5.8–6.8) sodium acetate (pH 5.4) buffer supplemented with 140 mM KCl and 20% PEG-400 to a final concentration of 3 μM and annealed rapidly (heated to 90 °C for 5 min and then snap-cooled on ice) to facilitate intramolecular folding prior to each experiment. CD spectra were registered at 20 °C using a Chirascan spectrophotometer (Applied Photophysics, Surrey, UK) in quartz cuvettes of 1 cm path in triplicate and averaged after baseline correction. The CD amplitude at 288 nm was plotted as a function of pH, normalized to a maximum value of 100%, and fitted to the following equation:

CD^norm^_pH*i*_ = CD^norm^_pH8_ + (100 − CD^norm^_pH8_)/(1 + 10^[pH*_i_*^^−pH_1/2_^^]×Slope^) (1)

where CD_pH8 is a residual amplitude of the unfolded C-rich oligonucleotide at the iM-specific maximum, slope indicates cooperativity, and pH1/2 is the inflection point (transition point).

The iM-destabilizing/G4-stabilizing naphthalene diimide QN-302 [38] was obtained from MedChemExpress (Monmouth Junction, NJ, USA), and the iM-destabilizing heliomycin derivative LCTA-2614 (initially referred to as 8e) was synthesized following the published procedure [37]. The purity of the sample was 99% (HPLC, Figure S1). The structure was confirmed by comparing the ^1^H and ^13^C NMR spectra (Figure S2) to those reported previously [37]. To verify the iM-destabilizing effects on the promoter/UTR-derived oligonucleotides, QN-302, LCTA-2614, or blank DMSO-containing solutions were mixed with oligonucleotide solutions in 140 mM KCl- and 20% PEG-containing sodium phosphate buffers (pH 6.0–6.8), to a final concentration of QN-302/ LCTA-2614 of 20 μM (0.5% DMSO). The samples were reannealed rapidly, CD spectra were measured at 20 °C, and CD-melting curves were obtained by CD monitoring at 288 nm upon temperature ramping at a rate of 1 °C/min. The melting temperatures were determined by fitting the experimental data to a sigmoid curve with slope using ProData software (Applied Photophysics, Surrey, UK).

### 2.2. Plasmids

The previously tested reporter vector CMV-FLuc [40], encoding Firefly luciferase (FLuc) under full-length CMV promoter, was obtained from Promega (Madison, WI, USA) and used as a control plasmid in luciferase assays. The reporter vector pRL-TK (GenBank: AF025846), encoding Renilla luciferase, was obtained from Addgene (Watertown, MA, USA) and used as a reference plasmid.

To obtain RHEBp/PIM1p/JUNp/EGR1p plasmids, iM/G4-sequences with 5 bp flanks from respective promoters were cloned into CMV-FLuc after the NdeI site of the CMV enhancer-promoter (350 bp from TSS) using polymerase incomplete primer extension (PIPE) method [41] with vector-binding primers pGLpipeF (5′-GGTAAGACACGACTTATCGCCACTG) and pGLpipeR (5′-AAGTCGTGTCTTACCGGGTTGGACTC) and the following insert-binding primers (underlined, iM/G4 fragments; uppercase, genomic flanks):

JUNp-for: 5′-ggggaccggggaagagagggCCGAGccaagtacgccccctattgac
JUNp-rev: 5′-tcttccccggtcccctccccGGGCCcatatgatacacttgatgtactg
PIM1p-for: 5′-tcacgccccgcgcccctccccCTCCAccaagtacgccccctattgac
PIM1p-rev: 5′-gggcgcggggcgtgaggggAGGCGcatatgatacacttgatgtactg
EGR1p-for: 5′-ggcccgactcgccctcgcccccgCTCTCcaagtacgccccctattgac
EGR1p-rev: 5′-agggcgagtcgggccggggTTCTAcatatgatacacttgatgtactg
RHEBp-for: 5′-gccccgcccccccctccccgCCTCCcaagtacgccccctattgac
RHEBp-rev: 5′-gaggggggggcggggCTGCCcatatgatacacttgatgtactg

To obtain the MUTp plasmid, the non-iM/G4-forming sequence, obtained by mutating the EGR1p sequence with the G4 killer tool of the DNA Analyzer, was cloned into the CMV-FLuc using the same method with the following insert-binding primers:

MUT-for: 5′-ggctcgactcgctctcgcaacagCTCTCcaagtacgccccctattgac
MUT-rev: 5′-agagcgagtcgagccgtgtTTCTAcatatgatacacttgatgtactg

To obtain RHEBu and EGR1u plasmids, iM/G4 sequences with 11 bp flanks from respective UTRs were cloned into CMV-FLuc after the CMV promoter using the HindIII/XhoI sites (33/39 bp from TSS). The inserts with sticky ends were obtained by annealing the following synthetic oligonucleotides (underlined, iM/G4 fragments; uppercase, flanks):

EGR1u-f: 5′-tcgagcgCAACTGTGTcccctgcagctccagccccgggctcacccccccgccccGACACCAGCTCa
EGR1u-r: 5′-agcttGAGCTGGTGTCggggcgggggggtgcagcccggggctggagctgcaggggACACAGTTGCGc
RHEBu-f: 5′-tcgagCGGCCGGTCACGTgggcgtgttgtgggggggaggggCGCCGCCGCGCGGa
RHEBu-r: 5′-agcttCCGCGCGGCGGCGcccctcccccccacaacacgcccACGTGACCGGCCGc

The resulting plasmids were transformed into *E. coli* Top10 competent cells, and the cells were grown overnight on an ampicillin-containing agar at 37 °C. Colony PCR screening was performed using insert-overlapping forward primers and the reverse primer FLuc-rev (5′-CAGGAACCAGGGCGTATCTC). Selected clones were incubated overnight in an ampicillin-containing medium. Then, the plasmid was isolated using the Plasmid Miniprep 2.0 kit (Eurogen, Moscow, Russia), and its sequence was verified by Sanger sequencing using the following primers: FLuc-for (5′-GGGTCATTAGTTCATAGCCCATAT) and FLuc-rev (CAGGAACCAGGGCGTATCTC). For each plasmid, up to 3 clones were selected and verified until the perfect sequence was found (Figure S3 for EGR1u and RHEBu, Figure S4 for PIM1p and JUNp, and Figure S5 for EGR1p, RHEBp, and MUTp).

### 2.3. Cell Culture, Toxicity Assays, and Dual Luciferase Assays

Human embryonic kidney (HEK-293) immortalized cells (ATCC, Manassas, VA, USA, catalog number CRL-1573) were cultured in a humidified 5% CO_2_ incubator at 37 °C, tested for mycoplasma contamination, and grown in 96-well culture plates in Dulbecco’s Modified Eagle Medium (DMEM) (Paneco, Moscow, Russia) supplemented with a 1% penicillin/streptomycin mixture (Paneco, Moscow, Russia), 2 mM glutamine (Paneco, Moscow, Russia), and 10% fetal bovine serum (Capricorn Scientific, Ebsdorfergrund, Germany) to 60% confluency. Then, cells were transfected with a 1:1 mixture of two plasmids, pRL-TK (reference) and CMV-FLuc (control) or its iM/G4-harboring derivative (50 ng each) using Lipofectamine-3000 (Thermofisher Scientific, Waltham, MA, USA), following the manufacturer’s protocol.

Three hours after transfection, the cells were washed with Ca^2+^-free and Mg^2+^-free Dulbecco’s phosphate-buffered saline (PBS) and then incubated for 7 h in (i) blank PBS (5 mM KCl, 138 mM NaCl, and 8 mM Na_2_HPO_4_/NaH_2_PO_4_, pH 7.4) with or without ionophores (valinomycin and nigericin, each at a final concentration of 10 μM), (ii) acidified PBS (pH 6.5) with 10 μM ionophores, or (iii) 20 μM LCTA-2614/QN-302 solution in PBS (pH 7.4). Each solution also contained DMSO at a final concentration of 0.5% from ionophore or LCTA-2614/QN-302 stock solutions, and 0.5% was also added to blank PBS. The experiments were performed in two series: (A) blank versus blank with ionophores versus acidification; (B) blank versus LCTA-2614 versus QN-302, each at least in 3 biological repeats (different days). Subsequent comparison of the two control groups (ANOVA for blank PBS with and without ionophores) revealed no significant differences, so these controls were eventually analyzed together. After 7 h of incubation, the cells were washed with PBS and treated with Passive Lysis Buffer from Dual-Luciferase Reporter Assay System (Promega, Madison, WI, USA).

The incubation time after transfection and LCTA-2614/QN-302 concentrations were selected to ensure sufficient reporter expression while avoiding pronounced cytotoxic effects. For that, the toxicity of LCTA-2614/QN-302 toward HEK-293 cells was tested prior to the main assays using Alamar Blue reagent (Thermo Fisher Scientific, Waltham, MA, USA) following the manufacturer’s protocol. To evaluate cell viability, fluorescence at 590 nm upon excitation at 560 nm was measured using a plate reader Infinite 200 PRO (Tecan, Männedorf, Switzerland).

Dual luciferase assays were performed using the Dual-Luciferase Reporter Assay System (Promega) following the manufacturer’s instructions. Luminescence measurements after the addition of FLuc substrate-containing reagent (FLuc signal at 560 nm) and RLuc substrate-containing Stop & Glo^®^ Reagent (RLuc signal at 480 nm) were performed using an Infinite 200 PRO reader (Tecan, Switzerland). Variance in the RLuc signal within each biological repeat was analyzed to verify reasonable consistency in transfection efficiency. For each CMV-FLuc derivative (ERG1u/p, RHEBu/p, JUNp, or PIM1p), co-transfected with RLuc-encoding pRL-TK, the RLuc signal in 3 technical repeats was compared to that obtained for unmodified CMV-FLuc/pRL-TK co-transfection (CNTR). The sample was excluded from further analysis if the RLuc signal difference between this sample and CNTR exceeded 20%. Otherwise, 3 technical repeats were averaged, and the FLuc/RLuc ratio was normalized by that of CNTR from the same transfection series (the same biological repeat): FLuc/RLuc^norm^ (%) = 100 × (FLuc/RLuc^sample^)/(FLuc/RLuc^CNTR^). If the standard deviation of the 3 technical repeats exceeded 20% in CNTR, the whole series was excluded. The resulting normalized FLuc/RLuc signal ratio values were compared for different CMV-FLuc derivatives (ERG1u/p, RHEBu/p, JUNp, or PIM1p) and incubation conditions (control, acidified PBS, or PBS supplemented with LCTA-2614/QN-302), and the significance of the differences between the derivatives/conditions was verified by analysis of variation (ANOVA) with post hoc Dunnett’s tests.

### 2.4. Molecular Modeling

The 3D model of QN302 was created using the built-in molecular editor of the docking software Molsoft ICM-Pro version 3.9-2 [42]. The lowest-energy conformations were determined using the Monte Carlo method. The MMFF force field was used to describe the interatomic interactions. The electron density distribution was determined by density functional theory (DFT) calculation using the hybrid functional B3LYP/6-311G++ Pople basis set. Then, the Merz–Singh–Kollman scheme was applied to the electron density distribution obtained for calculating the grid for the electrostatic potential fitting with the following parameters: (6/41 = 10)—the number of surfaces around the atoms and (6/42 = 17)—the density of test points on these surfaces. The Restrained ElectroStatic Potential (RESP) method was applied to the fitting of the grid obtained in the previous step for the calculation of the partial atomic charges. All quantum mechanics simulations were carried out using the Gaussian 16 program [43].

The intramolecular iM model was built based on the reported strategy [9] using Sybil-X software, version 2.1 (Certara, Radnor, PA, USA). First, to create the iM core, the coordinates for eight cytosine–cytosine pairs were obtained from the reported tetramolecular iM model (PDB 1YBL). Then, the loops were added step-wise, and at each step, MM-optimization was carried out using the SYBYL X and Powell method with the following settings: partial charges and parameters for interatomic interactions were from the Amber7ff02 force field, a non-bonded cut-off distance was set to 8 Å, a distance-dependent dielectric function was applied, the number of iterations was equal to 500, the simplex method was used in the initial optimization, and the energy gradient convergence criterion was 0.05 kcal/mol/Å.

For docking, the iM and QN-302 were converted into ICM objects following the ICM method (the molecular models were described using internal coordinates as variables). The parameters needed for interatomic energy calculation were taken from ECEPP/3 and from Merck Molecular Force Field (MMFF) for the QN-302 atoms. The partial charges of the iM atoms were also taken from ECEPP/3.

The models of the QN-302-iM complexes obtained from docking were verified by molecular dynamics (MD) simulations using Amber 24 software [44]. The influence of the solvent was simulated using the OPC3 water model. A rectangular box and periodic boundary conditions were used in the simulation. The space between the models and the periodic box wall was at least 15 Å. K^+^ and Cl^−^ ions were used for the creation of the ionic environment. The parameters needed for interatomic energy calculation were taken from the force fields OL15 (iM) and gaff2 (QN-302). At the beginning of the computation, the complexes were optimized in two stages. First, the location of the solvent molecules was optimized by using 1000 steps (500 steps of steepest descent followed by 500 steps of conjugate gradient). At this stage, the mobility of all solute atoms was restrained by a force constant of 500 kcal × mol^−1^ × Å^−2^. In the second stage, the optimization was performed without restrictions using 2500 steps (1000 steps of steepest descent and 1500 steps of conjugate gradient). Then, gradual heating to 300 K was carried out for 20 ps. To avoid spontaneous fluctuations at this point, weak harmonic restraints were applied with a force constant of 10 kcal × mol^−1^ × Å^−2^ for all atoms other than the solvent ones. The SHAKE algorithm was applied to constrain hydrogen-containing bonds, which allowed the use of a 2 fs time step. Scaling of 1–4 non-bonded van der Waals and electrostatic interactions was performed by using the standard Amber values. The cut-off distance for non-bonded interactions was set to 10 Å, and the long-range electrostatics were calculated using the particle mesh Ewald method. The MD simulations in the production phase were carried out using constant temperature (T = 300 K) and constant pressure (*p* = 1 atm) over 160 ns. To control the temperature, a Langevin thermostat was used with a collision frequency of 1 ps^−1^. The iM–QN-302 free energies were evaluated using the Molecular Mechanics/Generalized Born Surface Area (MM/GBSA) approach following the previously published procedure [45].

## 3. Results and Discussion

### 3.1. Genomic Intercalated Motifs (iMs) and Their pH-Dependent Folding

To select iMs whose impact on transcription may be comparable to or prevail over the effects of the opposing G4s (at least under iM-favoring conditions), we focused on HEK-293 cells, where both iMs and G4s have been mapped genome-wide by CUT&Tag under unified conditions [8]. We examined the regulatory regions of oncogenes and tumor suppressors because iM-favoring acidification is expected in the tumor microenvironment and can promote further transformation through transcriptional reprogramming [26]. This reprogramming is likely multi-step with complex secondary effects. However, we only aimed to verify the primary effect, i.e., transcription alteration due to iM folding as an early response to acidosis. Thus, we focused on immediate-early genes (IEGs) [46]. They are typically expressed transiently in response to external stimuli but show prolonged expression in tumor cells and model immortalized cells [47]. Apart from that, IEGs are expressed in normal neurons during synaptogenesis and are upregulated in pathologies associated with acidified brain tissue, such as neurodegenerative and psychiatric diseases characterized by chronic neuroinflammation [48,49]. In this regard, to select genes of interest, we overlapped available lists of IEGs [50] with confirmed oncogenes/tumor suppressors from OncoKB and TSGene 2.0 databases [51,52] and checked the matches with non-zero expression in HEK-293 cells, according to the Human Protein Atlas database [53], for the presence of promoter iM/G4 CUT&Tag peaks [8]. Finally, we checked each promoter region with iM peaks for the presence of C/G-rich sequences that are consistent with a consensus iM definition (C_3+_N_1–6_C_3+_N_1–15_C_3+_N_1–6_C_3+_) and excluded sequences with long central loops. Although biologically relevant, long-loop iMs may contain additional noncanonical elements and are suboptimal for proof-of-concept studies. To summarize, the genomic sequences analyzed in this study were selected based on the following criteria:

- Reproducible iM CUT&Tag peaks (iM CUT&Tag score ≥ 100) in HEK-293.
- The presence of four C_3+_-tracts within the CUT&Tag peak.
- The distance between the presumed iM and TSS is ≤500 nt.

**Figure 1:**
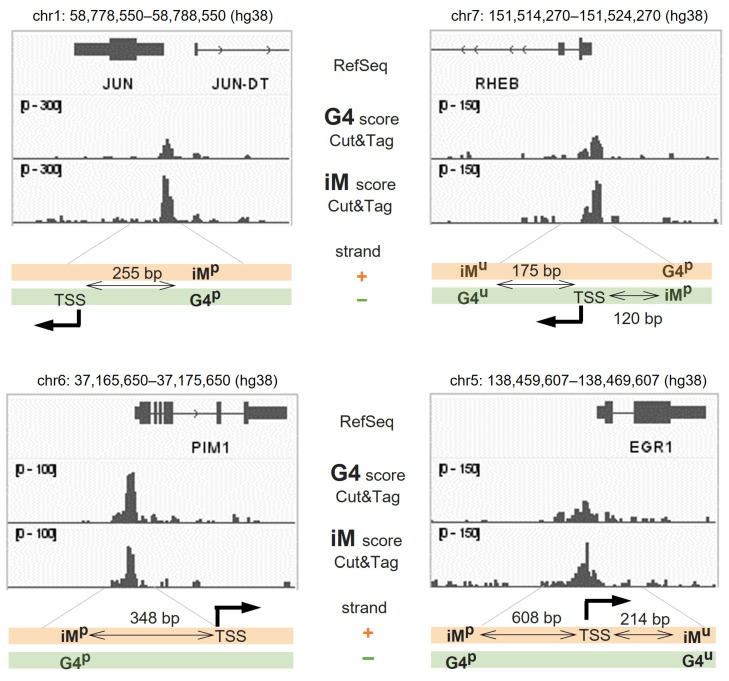
High-scoring iM/G4 sequences within CUT&Tag peaks in promoters and UTRs of human early response cancer-related genes.

The final list of genomic sequences that meet the above criteria included promoters/UTRs of a tumor suppressor gene *EGR1* (encodes an early growth response factor), oncogenes *JUN* (encodes a c-Fos binding component of the early response transcription factor) and *RHEB* (encodes an mTOR-activating Ras homolog), and a protooncogene *PIM1* (encodes a serine–threonine kinase from the PIM family). Promoters of *EGR1*, *JUN*, and *RHEB* showed higher iM CUT&Tag score than the G4 score, while in the case of *PIM1*, these scores were comparable (Figure 1).

Next, we examined the G/C-rich sequences from the CUT&Tag peaks in further detail to identify fragments with the highest iM-forming potential. For this, the CUT&Tag peaks were screened using a G4Hunter tool of the DNA Analyser [39] with a window of ≥25 nt, and the sequences with the most pronounced G/C skewness (G4Hunter score <−1.8 for stable iMs) were selected. *JUN* and *PIM1* promoters contained iMs of high predicted folding propensity in template and non-template strands, respectively. These iMs are hereafter referred to as JUNp (255 bp from TSS) and PIM1p (348 bp from TSS). The CUT&Tag peaks related to *EGR1* and *RHEB* spanned both promoters and UTRs. The respective top-scoring promoter iMs are referred to as EGR1p (non-template strand, 608 bp from TSS) and RHEBp (template, 120 bp from TSS). The top-scoring UTR iMs are referred to as EGR1u (non-template strand, 214 bp from TSS) and RHEBu (non-template strand, 175 bp from TSS).

**Figure 2:**
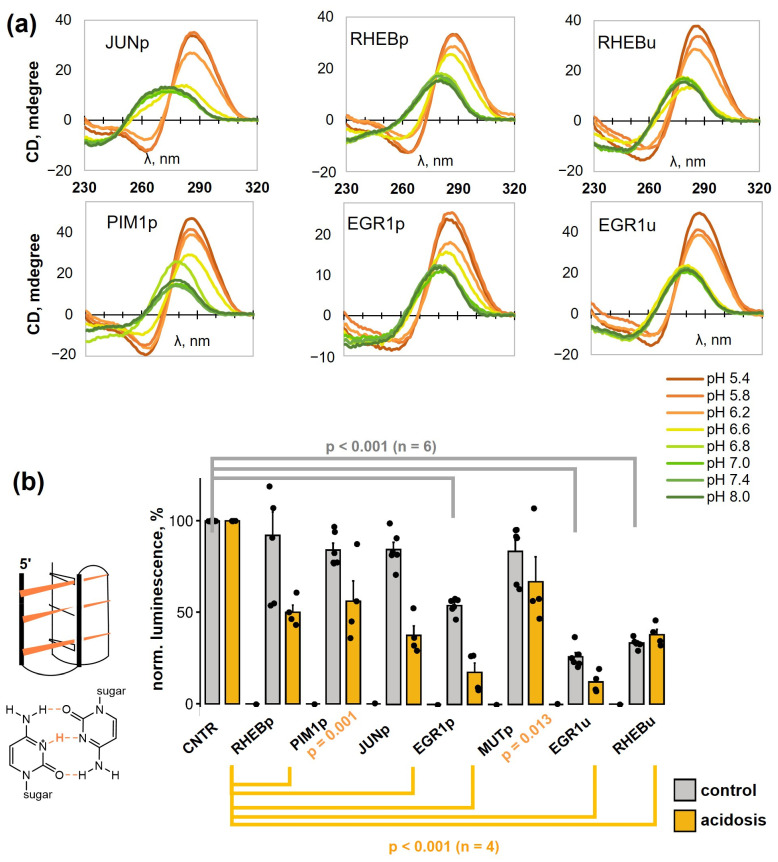
pH-Dependent folding of the genomic iMs and their impact on the transcription of the reporter gene. (**a**) CD-based verification of iM pH-dependence. Conditions: 3 μM iM in a 10 mM sodium phosphate (pH 5.8–8.0) or sodium acetate (pH 5.4) buffer, supplemented with 140 mM KCl and 20% PEG-400. (**b**) Schematic representation of intramolecular iM folding in mildly acidic media due to cytosine protonation (**left**) and DLA-based verification of iM effects on transcription (**right**). The DLA results are shown for HEK-293 cells transfected with the specified CMV-FLuc plasmid and the RLuc-encoding pRL-TK plasmid with subsequent incubation in native or acidic (pH 6.5) media. Normalized luminescence (mean ± SE) is the FLuc/RLuc signal ratio of the specified CMV-FLuc derivative normalized by that of CNTR. Sample size: 6 (control) or 4 (acidosis). Significance of FLuc regulation by the specified insertion compared to no insertion (CNTR) was verified by Dunnett’s test. Gray and yellow lines indicate highly significant (*p* < 0.001) regulation in control and acidic media, respectively. For significant and marginally significant (0.001 ≤ *p* ≤ 0.05) regulation, exact *p*-values are shown below the respective bars. Other changes are not statistically significant.

Prior to verifying acidosis-specific transcription regulation by these iMs, we characterized their pH-dependence in vitro using synthetic oligonucleotides that comprise core iMs and 5-nt genomic flanks. The iM folding was verified by circular dichroism (CD) spectroscopy. Prior to that, all oligonucleotides were annealed in buffered solutions of physiological ionic strength (140 mM KCl) supplemented with polyethylene glycol PEG-400 to partially mimic the crowded environment of the nucleus in the G-phase [54]. Recently, crowders PEG 400–1000 were shown to stabilize iMs through altering the configuration of the stacked C·C^+^ base pairs. In contrast, lower molecular weight crowders such as PEG-200 did not favor iM formation and were assumed to mimic the cellular microenvironment in the S-phase, when the iMs are depleted [55]. The CD spectra of the oligonucleotides at pH 5.4–8.0 are shown in Figure 2a. The iM-specific CD signature (a positive band at 288 nm) was evident in the spectra of all oligonucleotides in mildly acidic buffers. The pH-dependence of its amplitude was fitted to a sigmoid curve in each case (Figure S6), which yielded the pH transition points, pH_1/2_ (Table 1). These values varied between 6.3 and 6.6, supporting the potential significance of the iMs in moderate-to-severe acidosis. Unlike the iM-forming C-rich oligonucleotides from Table 1, the complementary G-rich oligonucleotides had no significant pH-dependence, according to CD spectroscopy data (Figure S7). Their spectra contained signatures of mixed-topology or parallel-stranded G4s (the positive bands at 295 and/or 265 nm); however, these spectra were almost similar at pH 5.4 and 8.0, which excludes sensitivity to acidosis.

To summarize this part, we showed that C-rich sequences from the promoters and UTRs of human early response genes are potentially responsive to pH alterations in the biologically relevant pH range (6.5–7.4). We could not fully recapitulate intracellular conditions in CD assays, so the fraction of folded iMs might be underestimated. However, it is sufficient to interfere with transcription at least in the case of acidosis. Thus, we next designed plasmids for investigating the effects of these iMs on the expression of the reporter gene (luciferase). Reporter plasmids do not enable thorough verification of context-dependent transcription regulation. For instance, the 3D genome packaging and the potential interplay between the tested sequences and distant cis-regulatory elements cannot be explored using luciferase assays. Moreover, such assays do not allow for distinguishing between transcriptional and translational regulation. However, the latter limitation appears to be non-crucial in the case of iMs, considering the generally low stability of RNA iMs, which argues against their interference with translation [5].

### 3.2. Regulation of Reporter Transcription by Genomic iMs in Native and Acidic Media

**Figure A1:**
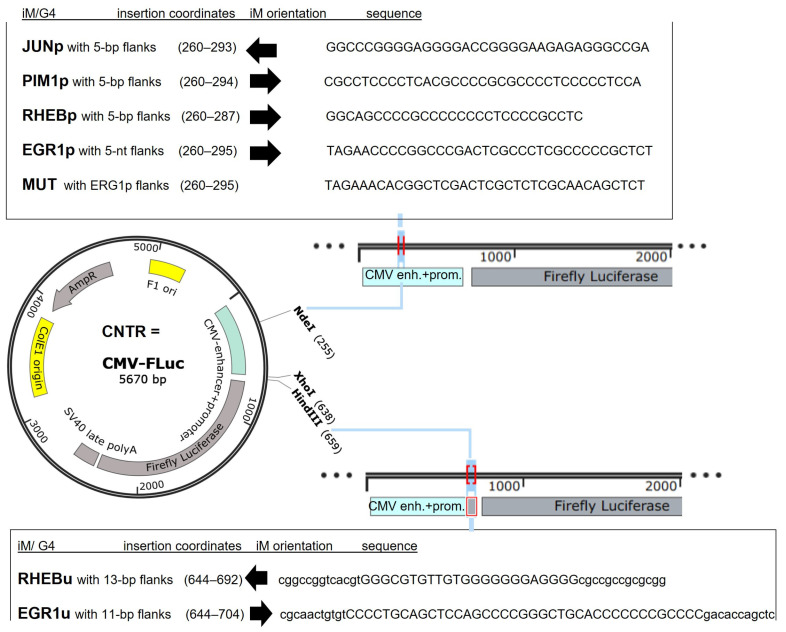
iM-harboring plasmids used for luciferase assays (schematic representation). The plasmids were obtained based on the previously reported control one (CNTR) by incorporating promoter iMs after the NdeI restriction site or UTR iMs at the XhoI/HindIII sites.

We designed a set of plasmids encoding the *Firefly* luciferase (FLuc) under the CMV promoter with iM-containing insertions in the middle of the promoter region, 350 bp upstream TSS, which is close to the mean iM-TSS distance in the human promoters of interest (Figure 1), or in the 5′-UTR, 38 bp downstream TSS. The insertions contained core iMs/G4s (underlined sequences in Table 1 and their complements) with 5–13 bp flanks (Figure A1). Their orientation in the resulting construct was similar to that in the human genome (Figure 1): iMs JUNp and RHEBu were in the template strand, and iMs RHEBp, PIM1p, EGR1u, and EGR1p were in the non-template strand. The control CMV-FLuc-encoding plasmid (CNTR) contained no insertions, and another control (MUT) harbored a mixed sequence (non-iM/non-G4) fragment. It was obtained from EGR1p by multiple mutations using the G4Killer tool [39].

The effects of the insertions on the FLuc level were assessed in HEK-293 cells—initially, under normal conditions (native pH). To account for potential variations in transfection efficiency or unspecific effects of acidosis on transcription/translation, co-transfections with an iM-free plasmid encoding an FLuc-orthogonal reporter, *Renilla* luciferase (RLuc), were used. For each CMV-FLuc-based plasmid, a luciferase ratio (FLuc/RLuc) in HEK-293 cells was assessed 10 h after co-transfection using dual luciferase assays (DLA) and normalized to the FLuc/RLuc ratio obtained with the CNTR CMV-FLuc plasmid. The results are summarized in Figure 2b (gray bars).

Most of the promoter insertions, including the control one (MUT), had insignificant effects. The only exception was EGR1p, which caused a nearly 2-fold decrease in the FLuc/RLuc ratio. Interestingly, this iM is the closest to the canonical motif with short loops 1 and 3 and the slightly longer central loop [2]. However, in terms of the C^+^/C stacking efficiency evidenced by CD_288 nm amplitude (Figure 2), or the protonation efficiency summarized as pH_1/2_ (Table 1), it has no apparent advantages over other promoter iMs. The UTR insertions (EGR1u and RHEBu) also caused a pronounced (>50%) decrease in the FLuc/RLuc ratio. Importantly, it was unclear from the DLA results whether iMs or G4s accounted for the regulation.

To verify the contributions of the iMs, we next assessed FLuc levels in the transfected cells after their incubation at pH 6.5 (yellow bars in Figure 2b). The effect of MUTp remained marginal. In contrast, the effects of RHEBp, JUNp, EGR1p, and EGR1u became more pronounced at pH 6.5: all these insertions caused a significant decrease in the FLuc/RLuc ratio. The difference between native pH and pH 6.5 was hardly significant for RHEBp and PIM1p due to the substantial variance (*p* > 0.01), whereas for JUNp, EGR1p, and EGR1u it was large and significant (*p* < 0.01), supporting the iM contribution to FLuc downregulation. In contrast, RHEBu showed similar downregulation of FLuc in native and acidic media, arguing for the G4 contribution rather than the iM contribution. Importantly, this regulation might occur during translation (the RHEBu G4 motif from the non-template strand is present and probably folded in RNA). In contrast, for acidosis-sensitive promoter insertions and the EGR1u insertion, effects on translation can be excluded (the EGR1u iM from the non-template strand is unfolded in RNA [5]).

To summarize this part, a comparative analysis of DLA results for the transfected cells incubated under native and iM-favoring acidic conditions supported downregulation of the reporter transcription by iMs JUNp, EGR1p, and EGR1u. To further verify this point, we next considered iM-disfavoring conditions.

### 3.3. Altered Regulation of the Reporter Transcription in the Presence of the iM-Disrupting Ligands

In a search for iM-disfavoring conditions, we considered treatment with iM-binding small molecules [56]. The majority of such small molecules have been initially developed as G4 ligands, and their ability to discriminate these iMs and G4s has hardly been characterized in sufficient detail in vitro [57]. Notable exceptions include a heliomycin derivative LCTA-2614 [37,58], a naphthalene diimide derivative QN-302 (SOP-1812) [38] and bisindolylmaleimide derivatives (BIMs) [59]. LCTA-2614, the iM destabilizer (initially referred to as 8e [37]), is remarkable for its selectivity to iMs over G4s [37], while QN-302 and BIMs bind both structures with the opposite effects: iMs are destabilized, and G4s are stabilized [60,61]. We selected treatment with LCTA-2614 and QN-302 as model iM-disfavoring and iM-disfavoring/G4-favoring conditions, respectively. The incubation time in the presence of LCTA-2614/QN-302 was similar to that in acidic media (a 3 h incubation without treatment after transfection, followed by a 7 h treatment; 10 h in total), enabling direct comparison of the DLA results in “control”, “acidosis”, and “ligand” groups.

**Figure 3:**
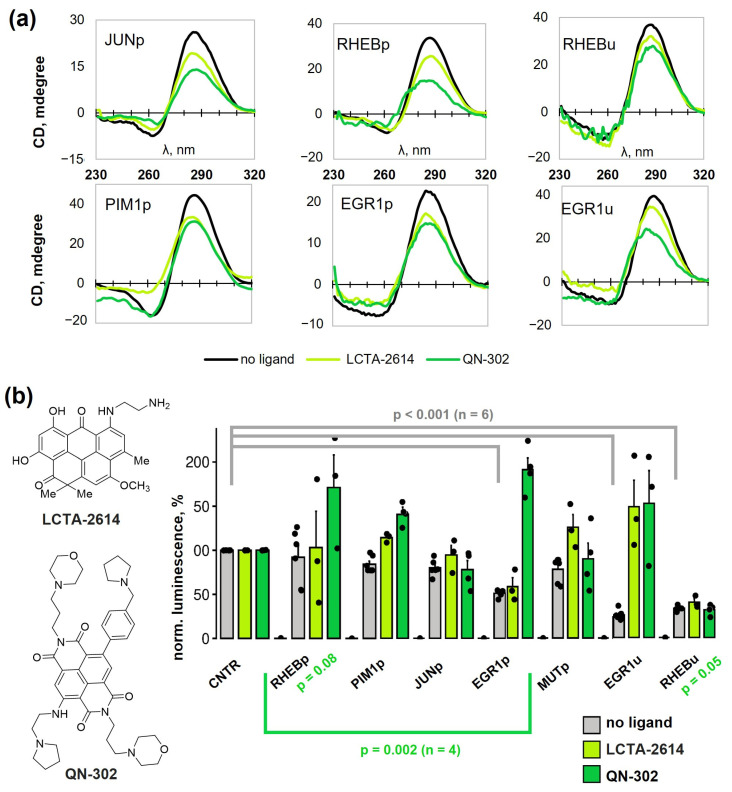
Ligand-induced destabilization of the genomic iMs and its impact on the transcription of the reporter gene. (**a**) CD-based verification of the iM destabilization by ligands LCTA-2614 and QN-302. Conditions: 3 μM iM and 20 μM ligand in 10 mM sodium phosphate buffer (pH 6.0) supplemented with 140 mM KCl and 20% PEG-400. (**b**) Structures of the ligands (**left**) and DLA-based verification of their effects on transcription (**right**). The DLA results are shown for HEK-293 cells transfected with the specified CMV-FLuc plasmid and the RLuc-encoding pRL-TK plasmid with subsequent incubation in the absence or in the presence of the ligands. Normalized luminescence (mean ± SE) is the FLuc/RLuc signal ratio of the specified CMV-FLuc derivative normalized by that of CNTR. Sample size: 6 (control), 3 (LCTA-2614), or 3–4 (QN-302). Significance of FLuc regulation by the specified insertion compared to no insertion (CNTR) was verified by Dunnett’s test. Gray and green lines indicate highly significant regulation in the absence and the presence of ligands, respectively. For marginally significant regulation, exact *p*-values are shown below the respective bars.

The concentrations of the ligands that are subtoxic but sufficient for the primary effects were selected based on cytotoxicity assays (Figure S3) and in vitro CD assays with the synthetic iMs. Previous evaluations of the ligands’ toxicity relate to prolonged incubation [37,38]. After a short-term incubation (7 h), the effects on HEK-293 were moderate (30% ± 10% reduction in viability) even for high ligand concentrations (40 μM). At a concentration of 20 μM, both ligands had minor (~20%) but significant (*p* < 0.05) effects (Figure S8), so this concentration was used in CD assays for verifying iM destabilization. As evident from Figure 3a, the amplitude of the iM-specific band at 288 nm decreased by up to 30% or 55% in the presence of 20 μM LCTA-2614 or QN-302, respectively, at pH 6.0, indicating iM destabilization.

To quantitatively characterize this destabilization, the CD-melting curves of the iMs in the absence and in the presence of the ligands were registered (Figures S9–S11), and the melting temperature differences (delta Tm) were calculated (Table S1). Consistent with the pH_1/2_ analysis (Table 1), the melting assays suggest that the iMs are folded at pH 6.0 and near-physiological temperatures but only partially folded at pH 6.4. The destabilizing effects of LCTA-2614 were mostly minor (up to −8 °C) at pH 6.0–6.4 and moderate (up to −10 °C) at pH 6.8. QN-302 outperformed LCTA-2614 in most cases, causing substantial (up to −16 °C) destabilization at pH 6.0–6.4, and eliminated any signs of iM formation at pH 6.8. We conclude that the selected subtoxic ligand concentration is sufficient (at least with QN-302) to exclude iM formation in native intracellular media.

The impacts of LCTA-2614 and QN-302 on FLuc levels in the transfected HEK-293 cells incubated at native pH are summarized in Figure 3b. In contrast to acidification, the ligands either upregulated FLuc or caused no changes. The impact of QN-302 (if any) was superior or similar to that of LCTA-2614. These results agree qualitatively with in vitro assays and are consistent with the presumed opposite effects of promoter iMs and G4s on transcription. Taken together, the DLA results shown in Figure 2 and Figure 3 can be interpreted as follows.

RHEBp and PIM1p were inactive but became weak transcription repressors in acidosis and weak enhancers with QN-302 due to iM folding and G4 folding, respectively. JUNp was only active as a repressor in acidosis, likely due to iM folding, while the contribution of the opposing G4 was minor, consistent with the JUNp G4:iM ratio in CUT&Tag peaks (Figure 1). EGR1p, the only promoter repressor functional under native conditions, became the strongest repressor in acidosis and the strongest activator with QN-302. These observations enable us to assign the repressive effects of the promoter insertion to the iMs. Both UTR insertions repressed the reporter significantly, i.e., by 75 ± 6% (EGR1u) and 66 ± 3% (RHEBu) in the control assays (native conditions). However, RHEBu (G4 on the non-template strand) showed no condition-dependence, whereas the repressive effect of EGR1u (iM on the non-template strand) became more pronounced (nearly reached 90%) in acid and was reversed by LCTA-2614/QN-302, supporting the transcription-level regulation by the iM.

In summary, both G4s and iMs on a template strand can act as transcription roadblocks if positioned close to the TSS in 5′-UTR. Promoter iMs tend to repress transcription in luciferase assays. Their contributions are balanced by the transcription-activating promoter G4s under normal conditions, appear to dominate under pathological conditions (acidosis), and can be reversed by iM-disrupting ligands. Thus, small molecules with dual (G4-stabilizing and iM-destabilizing) activity may be particularly promising for transcriptional reprogramming. Such a reprogramming could be useful for combating acidosis-driven tumor invasion along with more direct strategies targeting carbonic anhydrase IX or various pH-regulating transporters [62].

In this regard, we examined QN-302 more closely. Its stabilizing effect on parallel-stranded G4s has been characterized in detail and elucidated using molecular modeling [63]. Briefly, the stabilization results from the efficient stacking of QN-302 onto the outer G-tetrad. In contrast, the basis for iM destabilization is poorly understood, partly due to the lack of crystal structures or NMR-based models of intramolecular iMs without additional structural elements, which limits in silico modeling. To address this issue, we employed the recently proposed approach by V.B. Tsvetkov [9] to generate a model of the intramolecular iM PIM1 based on the available tetramolecular structure (PDB 1YBL).

Our goal was to clarify how QN-302 binds to the iM in nearly neutral media and how this leads to iM destabilization. The pH-dependence of this destabilization (Table S1) must reflect the interplay between cytosine and QN-302 protonation. We assumed the cyclic amines of the *N*-alkyl pyrrolidine residues to be fully protonated in QN-302 because its pKa value is similar to that of most tertiary aliphatic amines (i.e., ~10 [64]). In contrast, the cyclic amines of the *N*-alkyl morpholine residues have reduced pKa values (~7 [65]) and may not be protonated in neutral media. However, they are stronger bases than the cyclic amines of the iM cytosine residues, even though cytosine pKa within the iM core must be close to the pH_1/2_ value (6.6 for PIM1), rather than to pKa of an isolated cytosine (~4.6) [66]. Thus, the two morpholino amines of QN-302 (and probably its single aromatic secondary amine) could compete with the cytosines for protons if they are brought into proximity to the iM core. 

**Figure 4:**
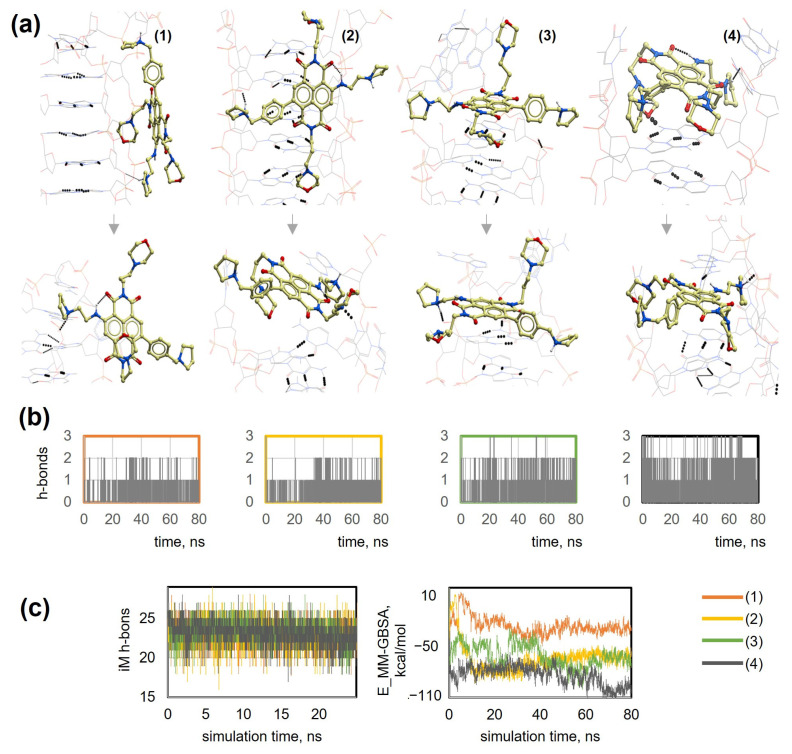
Molecular modeling of iM–QN-302 interactions. (**a**) Four representative conformations of PIM1p complexes with QN-302 obtained from docking (**top**) and subjected to 80 ns MD simulation (**bottom**). The ligand was docked to the iM minor groove (**1**), major groove (**2**), the cavity between the central loop and the terminal cytosine pair (**3**), and the cavity between the terminal pair and loops 1 and 3 (**4**). The black dotted lines indicate H-bonding. Ligand coloring: red, oxygen; blue, nitrogen; yellow, carbon. (**b**) Evolution of H-bonding between QN-302 and the iM throughout the MD simulation. (**c**) Evolution of the H-bonding within the iM during the first 25 ns of the simulation (**left**) and the iM–ligand free energy throughout the simulation (**right**). The energy was evaluated using the MM/GBSA approach.

We investigated this possibility by docking QN-302 onto the PIM1 iM. The docking procedure revealed the potential binding sites of four types: the iM minor groove (1), the iM major groove (2), the cavity between the central loop and the terminal cytosine pair (3), and the cavity between the terminal pair and loops 1 and 3 (4). Representative (high-scoring) complexes of each type are shown in Figure 4a, Top. Each of them was verified by MD simulation (Figure 4a Bottom). In complex (1), QN-302 barely maintained contact with the minor groove during the simulation. In complexes (2)–(4), at least one morpholino ring of QN-302 remained close to the iM core throughout the simulation. In complexes (3) and (4), where QN-302 was positioned near the outer cytosine pair, several intermolecular H-bonds were formed in most snapshots (Figure 4b). The cytosine pairs were mostly maintained, except for the minor disruption in complex (4) during the first 20 ns (Figure 4c Left). Importantly, this complex was most likely, according to the MM/GBSA energy estimations (Figure 4c Right). The competition between the morpholino ring and cytosines for protons in complex (4) could not be modeled explicitly. However, the final snapshots of the MD simulation illustrate the positioning of the morpholino ring consistent with such a competition. Thus, our results support the possibility of QN-302 interference with the terminal cytosine pair and provide a preliminary explanation for the iM disruption. Further verification of this preliminary explanation is needed, as is a comprehensive characterization of iM–ligand interaction modes using NMR spectroscopy and other techniques.

## 4. Conclusions

The iM-folding potential of C-rich sequences from promoters and UTRs of cancer-related IEGs was confirmed in vitro. These sequences were weak and strong transcription repressors in the reporter construct under native and iM-favoring (acidic) conditions, respectively. Their effects were mitigated or reversed by the iM-disfavoring conditions (ligands LCTA-2614 and QN-302). The findings suggest that despite the modest role in normal regulation, iMs could contribute significantly to transcriptional reprogramming in acidosis—e.g., during cancer progression. Our results encourage future studies of iM-disrupting ligands, such as LCTA-2614, as potential anticancer drugs, support the opposing regulatory roles of G4s and iMs, and underscore the particular therapeutic significance of the ligands with dual (iM-disrupting and G4-stabilizing) activity, such as QN-302.

## Figures and Tables

**Table 1:** Sequences of the genomic iMs, their G4Hunter scores, and transition points (pH_1/2_).

Code	Sequence	Score ^1^	pH_1/2_ ± 0.1
JUNp	CTCGG*CCC*TCTCTT*CCCC*GGT*CCCC*T*CCCC*GGGCC	−2.20	6.3
PIM1p	CGCCT*CCCC*TCACG*CCCC*GCG*CCCC*T*CCCC*CTCCA	−2.56	6.6
EGR1p	TAGAA*CCCC*GG*CCC*GACTCG*CCC*TCG*CCCCC*GCTCT	−1.92	6.5
EGR1u	TGTGT*CCCC*TGCAGCTCCAG*CCCC*GGGCTGCA*CCCCCCC*G*CCCC*GACAC	−1.80	6.5
RHEBp	GGCAG*CCCC*G*CCCCCCCC*T*CCCC*GCCTC	−3.50	6.6
RHEBu	CGGCG*CCCC*T*CCCCCCC*ACAACACG*CCC*ACGTG	−2.39	6.3

^1^ Scores are provided for the underlined sequences (italics, C_3+_ tracts).

## Data Availability

The original contributions presented in this study are included in the article/Supplementary Material. Further inquiries can be directed to the corresponding author.

## Supplementary Materials

The following supporting information can be downloaded at: https://www.mdpi.com/article/10.3390/biom16071038/s1. Figure S1: copy of HPLC chromatogram of LCTA-2614; Figure S2: NMR spectra of LCTA-2614; Figures S3–S5: plasmid sequencing; Figure S6: pH-Dependence curves of the iMs from UTR and promoter regions of IEGs.; Figure S7: CD spectra of G4s from UTR and promoter regions of IEGs; Figure S8: Impact of the ligands LCTA-2614 and QN-302 on the viability of HEK-293 cells.; Table S1: Melting temperatures of the iMs and the effects of the ligands; Figures S9–S11: Melting curves of the iMs in the absence and in the presence of the ligands.

## Abbreviations

The following abbreviations are used in this manuscript:

CD	Circular dichroism
CUT&Tag	Cleavage under targets and tagmentation
DLA	Dual luciferase assay
FLuc	*Firefly* luciferase
IEG	Immediate-early response genes
iM	Intercalated motif
G4	Guanine quadruplex
MM/GBSA	Molecular Mechanics/Generalized Born Surface Area
RLuc	*Renilla* luciferase
TSS	Transcription start site
UTR	Untranslated region
