# Supplementary material for: Intercalated Motifs Contribute to Transcription Regulation in an Acidosis Model
Source: Biomolecules. 2026 Jul 16;16(7):1038. doi: 10.3390/biom16071038 (PMC13406972; doi:10.3390/biom16071038)
Supplement: Supplementary file 1 [file biomolecules-16-01038-s001.zip › biomolecules-4379226-supplementary.pdf]

SUPPORTING INFORMATION

Intercalated motifs contribute to transcription regulation in an acidosis model

Varvara Sapozhnikova [1], Ekaterina Knizhnik [1], Dmitriy Shirokov [1,2], Yuri Khodarovich [3,4], Julia Khomyakova [1], Margarita Bogomiakova [1], Alexander Tikhomirov [5], Andrey Shchekotikhin [5], Anna Varizhuk [1,*] and Vladimir Tsvetkov [1,6,*]

1 Lopukhin Federal Research and Clinical Center of Physical-Chemical Medicine of Federal Medical Biological Agency, 119435 Moscow, Russia; annavarizhuk@gmail.com
2 K.I. Skryabin Moscow State Academy of Veterinary Medicine and Biotechnology, Moscow 109472, Russia; dmitry.a.shirokov@gmail.com
3 Shemyakin-Ovchinnikov Institute of Bioorganic Chemistry, Russian Academy of Sciences, 117997 Moscow, Russia; khodarovich@mail.ru
4 Research and Educational Resource Center for Cellular Technologies of The Peoples' Friendship University of Russia, 117198 Moscow, Russia; khodarovich@mail.ru
5 Gause Institute of New Antibiotics, 119021 Moscow, Russia; tikhomirov.chem@gmail.com (A.T.); shchekotikhin@mail.ru (A.S.)
6 A. V. Topchiev Institute of Petrochemical Synthesis RAS, 119991 Moscow, Russia; v.b.tsvetkov@gmail.com
* Correspondence: v.b.tsvetkov@gmail.com (V.T.) or annavarizhuk@gmail.com (A.V.).

Chromatogram
AST-866 C:\LabSolutions\Data\Gelio\TS V0708-03.lcd

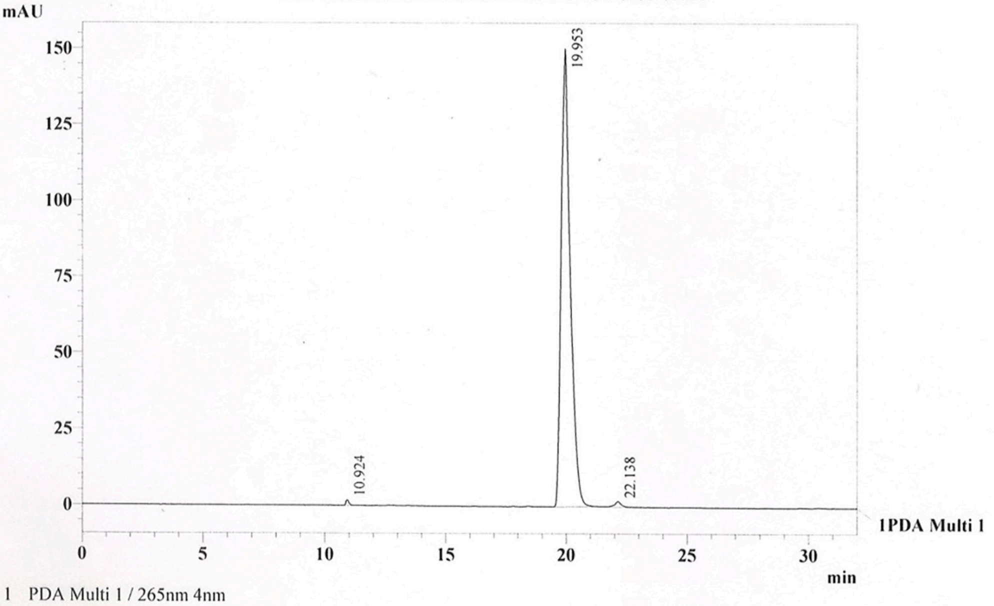

1 PDA Multi 1 / 265nm 4nm

PeakTable

PDA Ch1 265nm 4nm

Peak#	Ret. Time	Area	Height	Area %
1	10.924	14200	1732	0.357
2	19.953	3926948	150515	98.857
3	22.138	31195	1735	0.785
Total		3972343	153983	100.000

Method

<<LC Program>>

Time	Unit	Command	Value
0.10	Pumps	B.Conc	20
30.00	Pumps	B.Conc	90
33.00	Pumps	B.Conc	20
45.00	Controller	Stop	

Method Filename : FOS.lcm

Shimadzu LC-20AD; 2-System FOS, Colon Kromasil 100-C18,.size 5mkm, 4,6*250mm, N 86912
Elution: A - H3PO4 0.01M pH 2.6; B - MeCN, fl. 1,0 ml/min, loop 20mkl.

Figure S1. Copy of HPLC chromatogram of compound LCTA-2614.

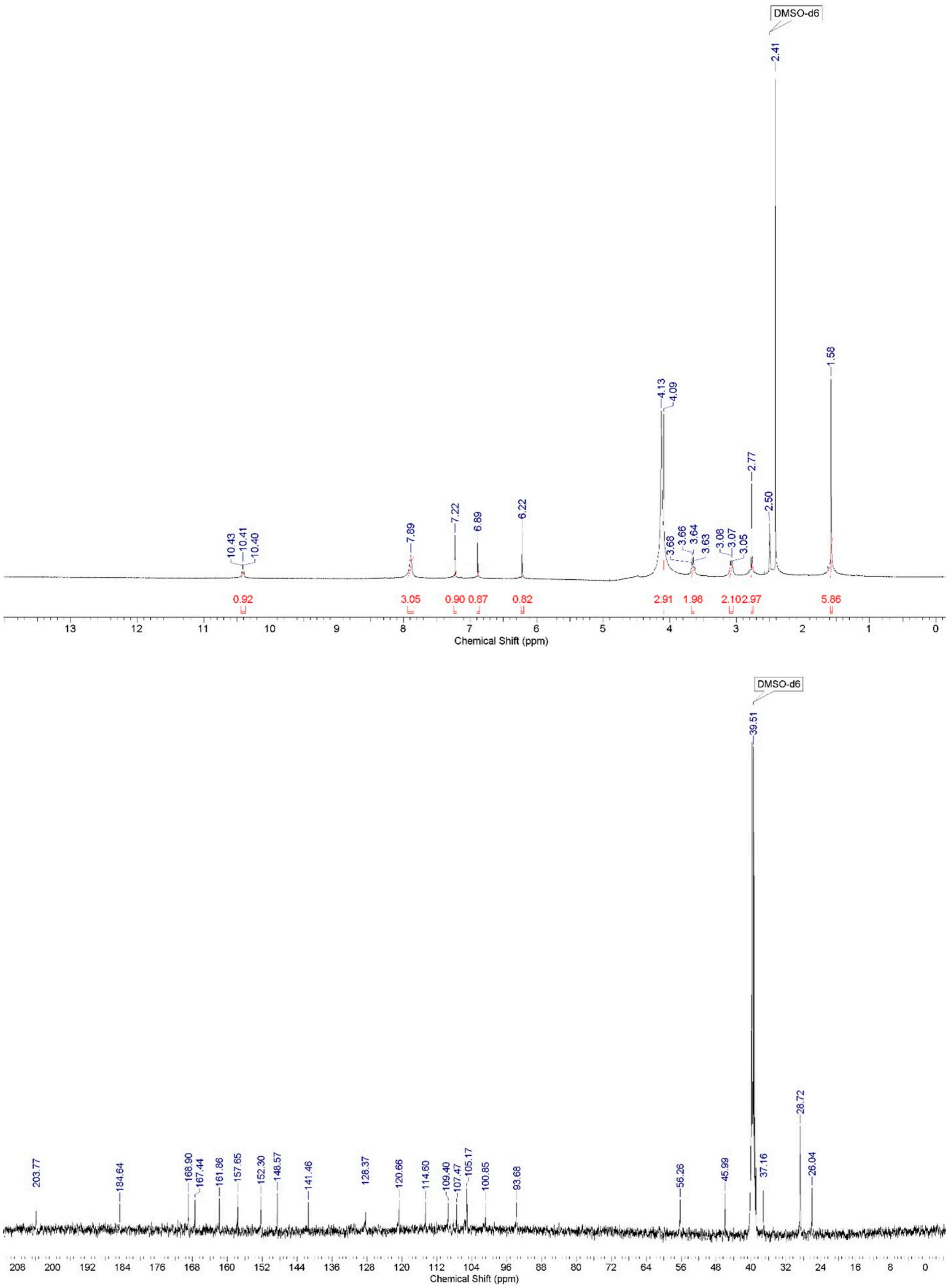

Figure S2. ^{1}H (top) and ^{13}C (bottom) spectra of compound LCTA-2614.

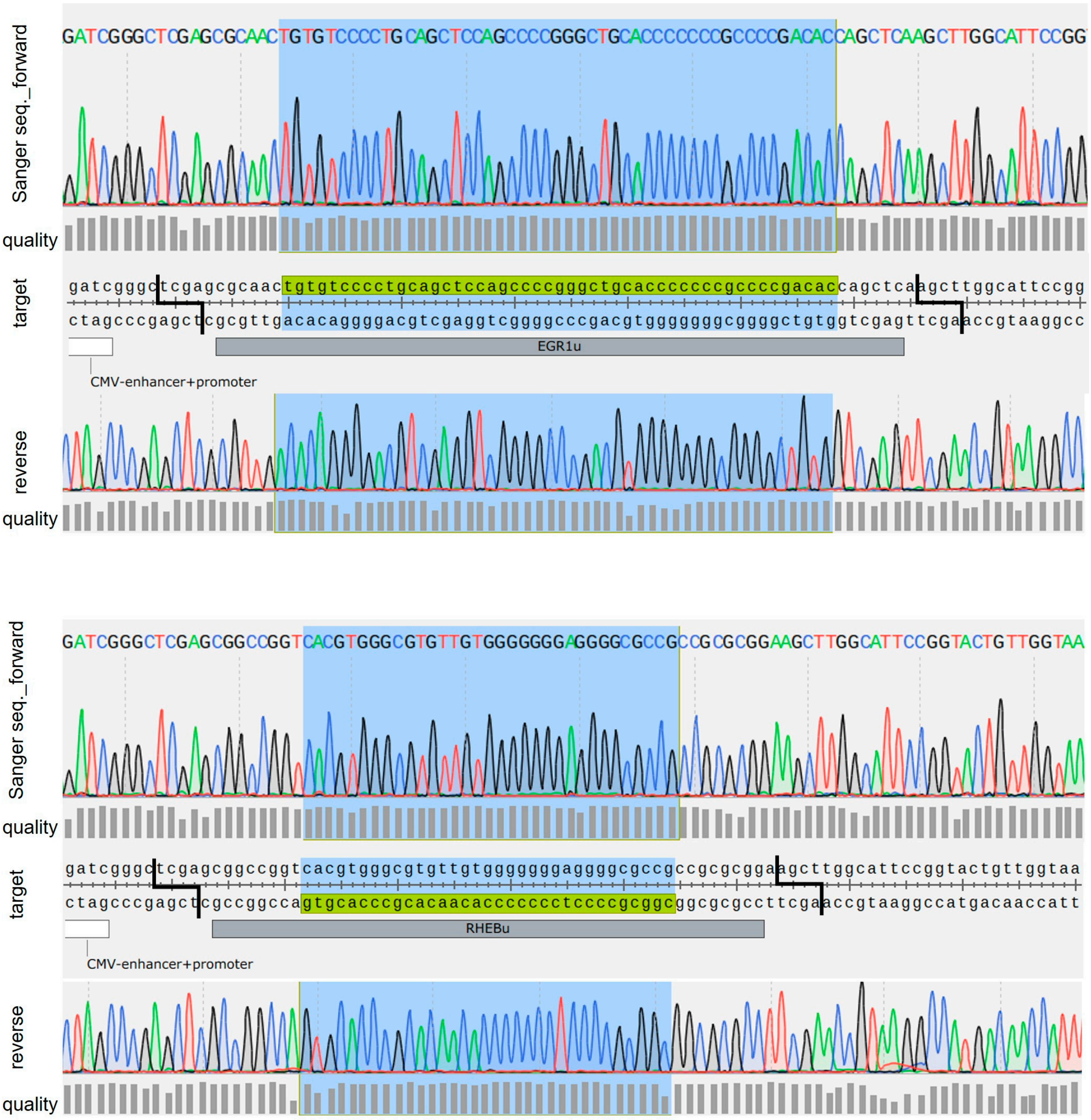

Figure S3. Sanger sequencing of plasmids with UTR insertions ERG1u and RHEBu. Gray, insertion; blue, core iM/G4-forming fragment with 5-nt flanks; yellow, the iM-forming sequence from Table 1; black lines, restriction sites XhoI/HindIII.

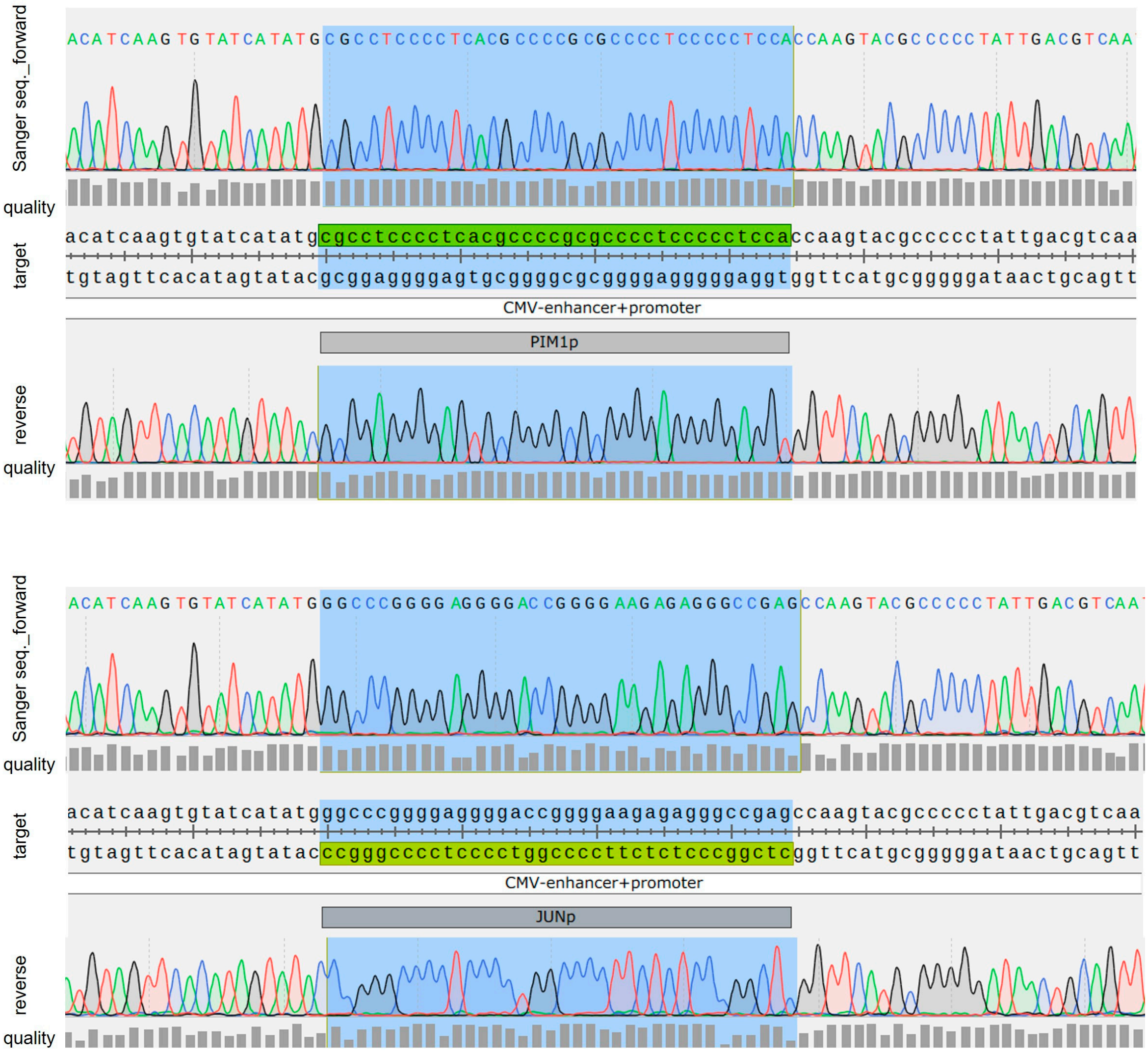

Figure S4. Sanger sequencing of plasmids with promoter insertions PIM1p and JUNp. Blue, core iM/G4-forming fragment with 5-nt flanks; yellow, the iM-forming sequence from Table 1.

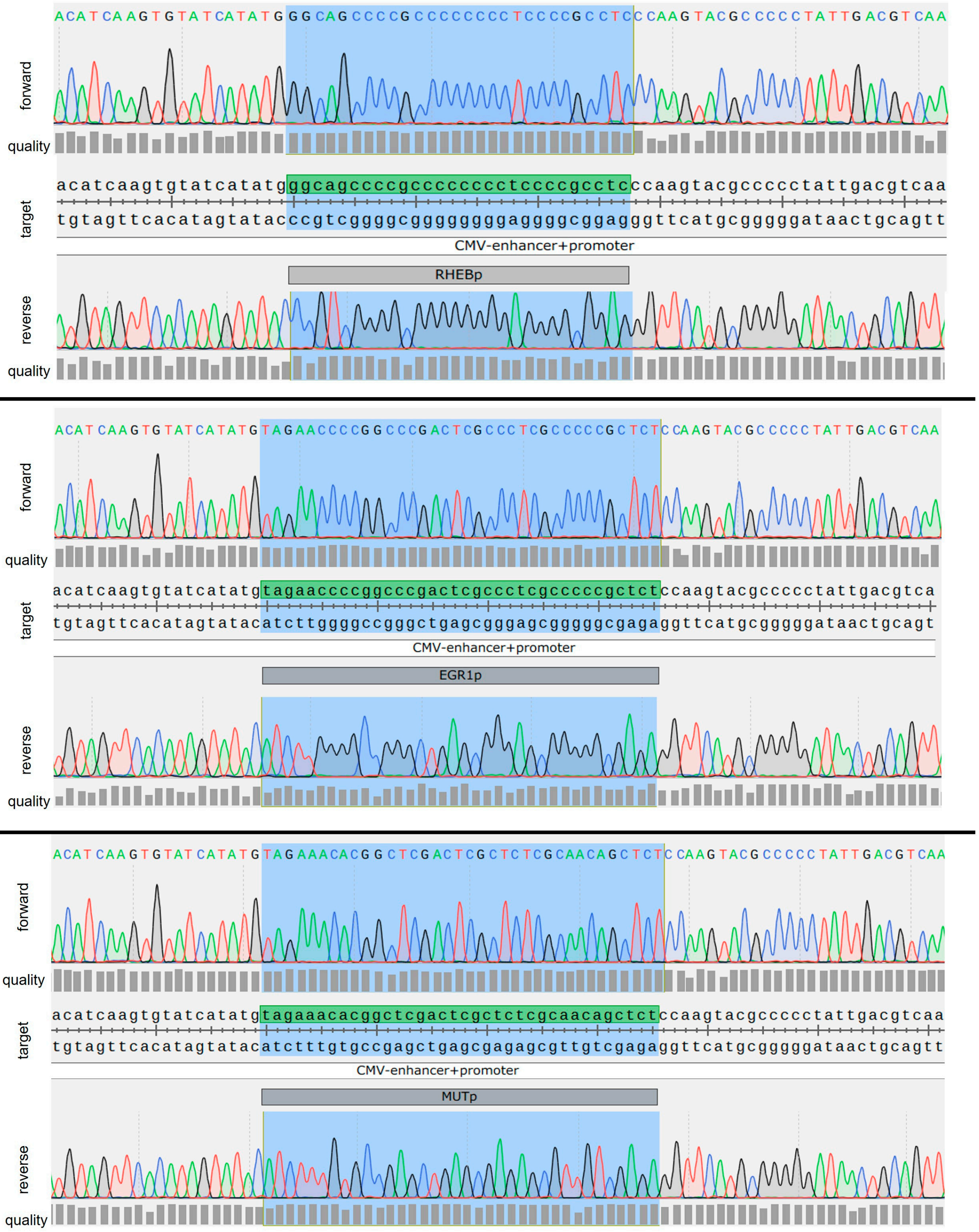

Figure S5. Sanger sequencing of plasmids with promoter insertions RHEBp, EGR1p and MUTp. Blue, core iM/G4-forming fragment with 5-nt flanks or its mutant; yellow, the iM-forming sequence from Table 1 or its mutant.

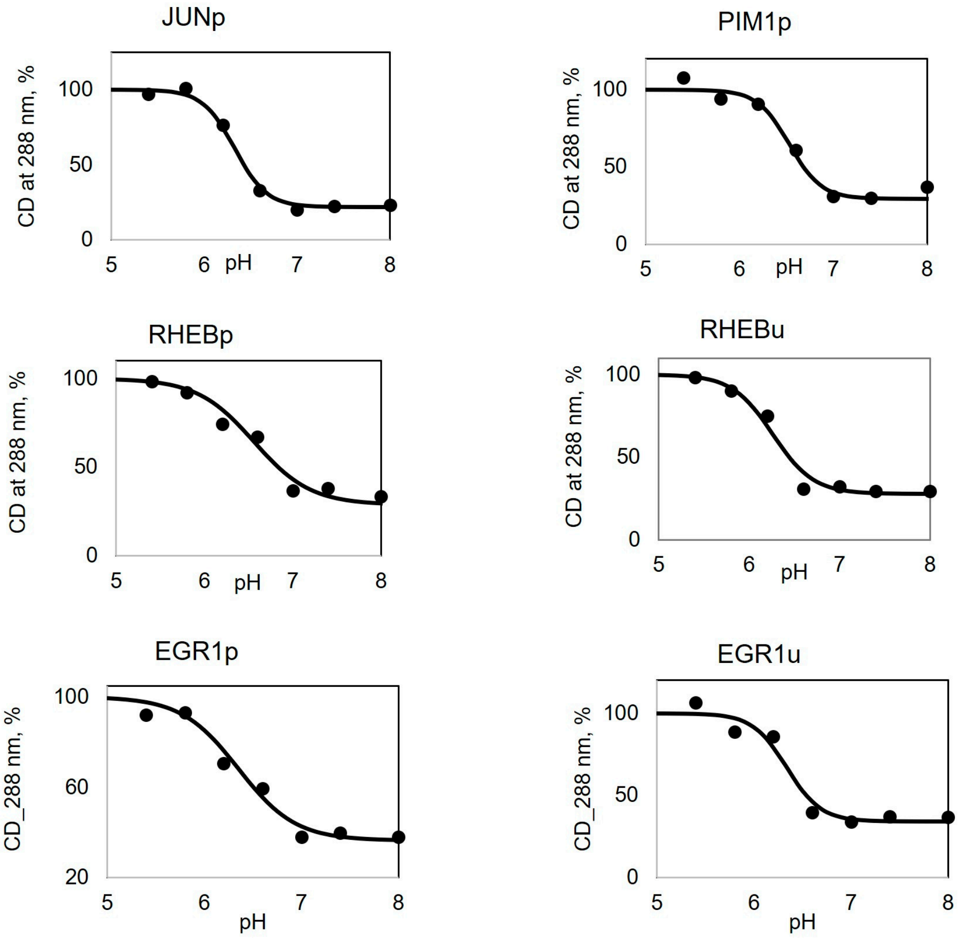

Figure S6. pH-Dependence curves of the iMs from UTR and promoter regions of IEGs. The curves were obtained by fitting the pH-dependence of CD amplitude of 3 μM iM samples in 10 mM sodium acetate or sodium-phosphate buffers, supplemented with 140 mM KCl and 20% PEG-400, to a sigmoid function.

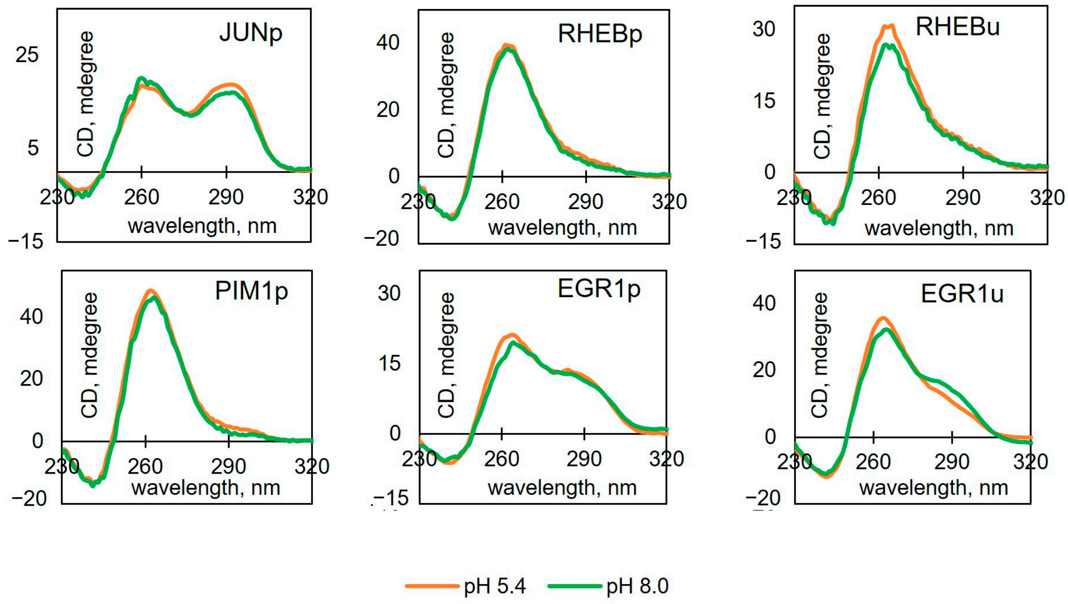

Figure S7. CD spectra of G4-forming oligonucleotides complementary to iMs from Table 1. Conditions: 3 µM G4 in a 10 mM sodium-phosphate (8.0) or sodium acetate (pH 5.4) buffer, supplemented with 140 mM KCl and 20% PEG-400.

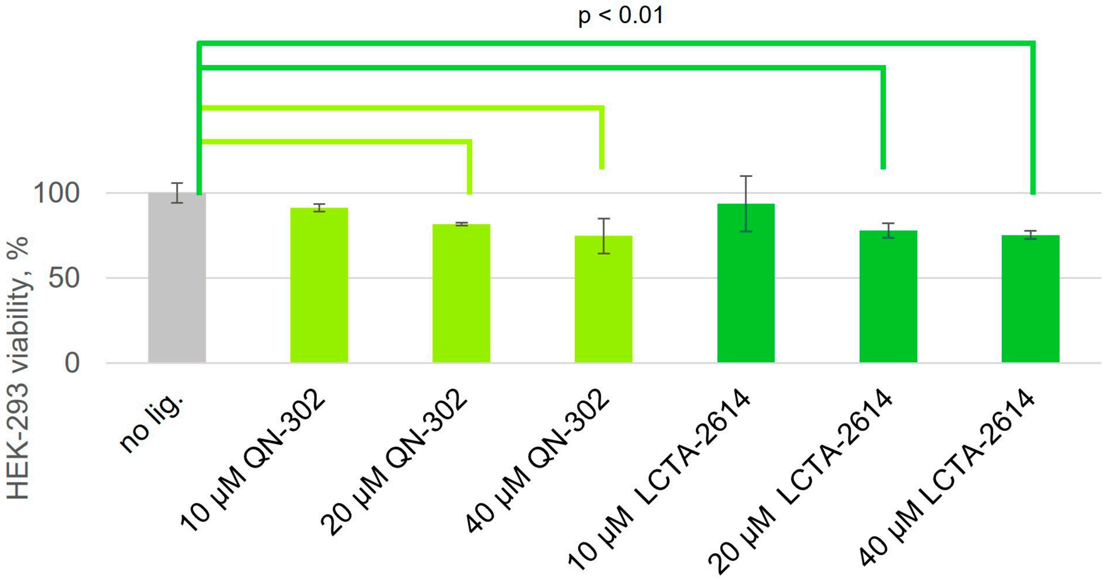

Figure S8. Impact of the ligands LCTA-2614 and QN-302 on the viability of HEK-293 cells.

Table S1. Melting temperatures of the iMs and the effects of the ligands

code	Tm, °C ± 1*			LCTA-2614_ΔTm, °C ± 2*			QN-302_ΔTm, °C ± 2*		
	pH 6.0	pH 6.4	pH 6.8	pH 6.0	pH 6.4	pH 6.8	pH 6.0	pH 6.4	pH 6.8
JUNp	57	22	18	1	-4	-6	-10	-10	no melt
PIM1p	51	24	22	-1	-1	-10	-16	-8	no melt
EGR1p	52	25	15	0	0	no melt	-7	-1	no melt
EGR1u	48	27	18	1	0	no melt	-3	-3	no melt
RHEBp	56	29	18	-8	-5	no melt	1	-5	no melt
RHEBu	58	28	no melt	1	-1	no melt	-7	-12	no melt

*from fitting to a sigmoid function

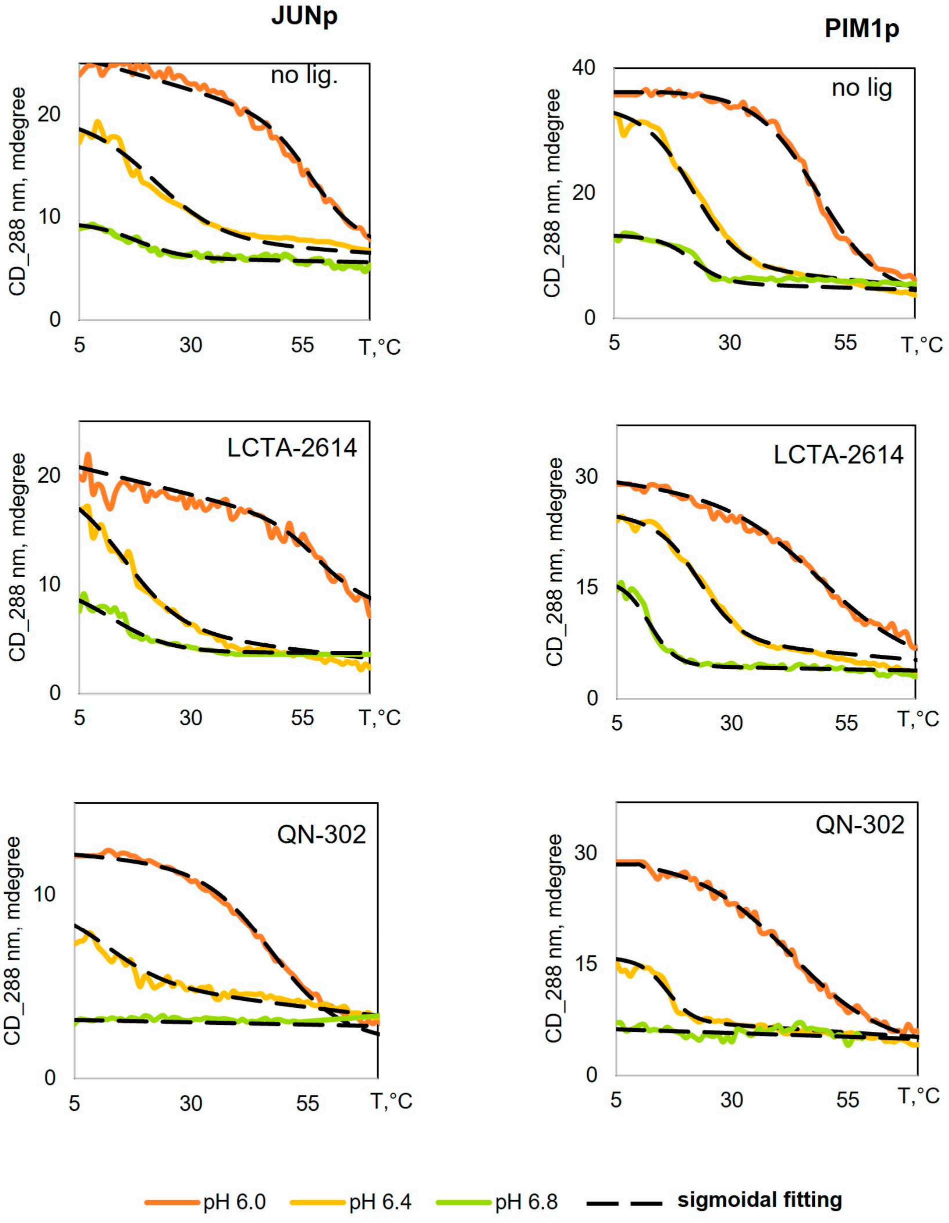

Figure S9. Melting curves of JUNp and PIM1p iMs in the absence and in the presence of the ligands. Conditions: 3 μM iM and 20 μM LCTA-2614/QN-302 in 10 mM sodium phosphate buffer supplemented with 140 mM KCl and 20% PEG-400.

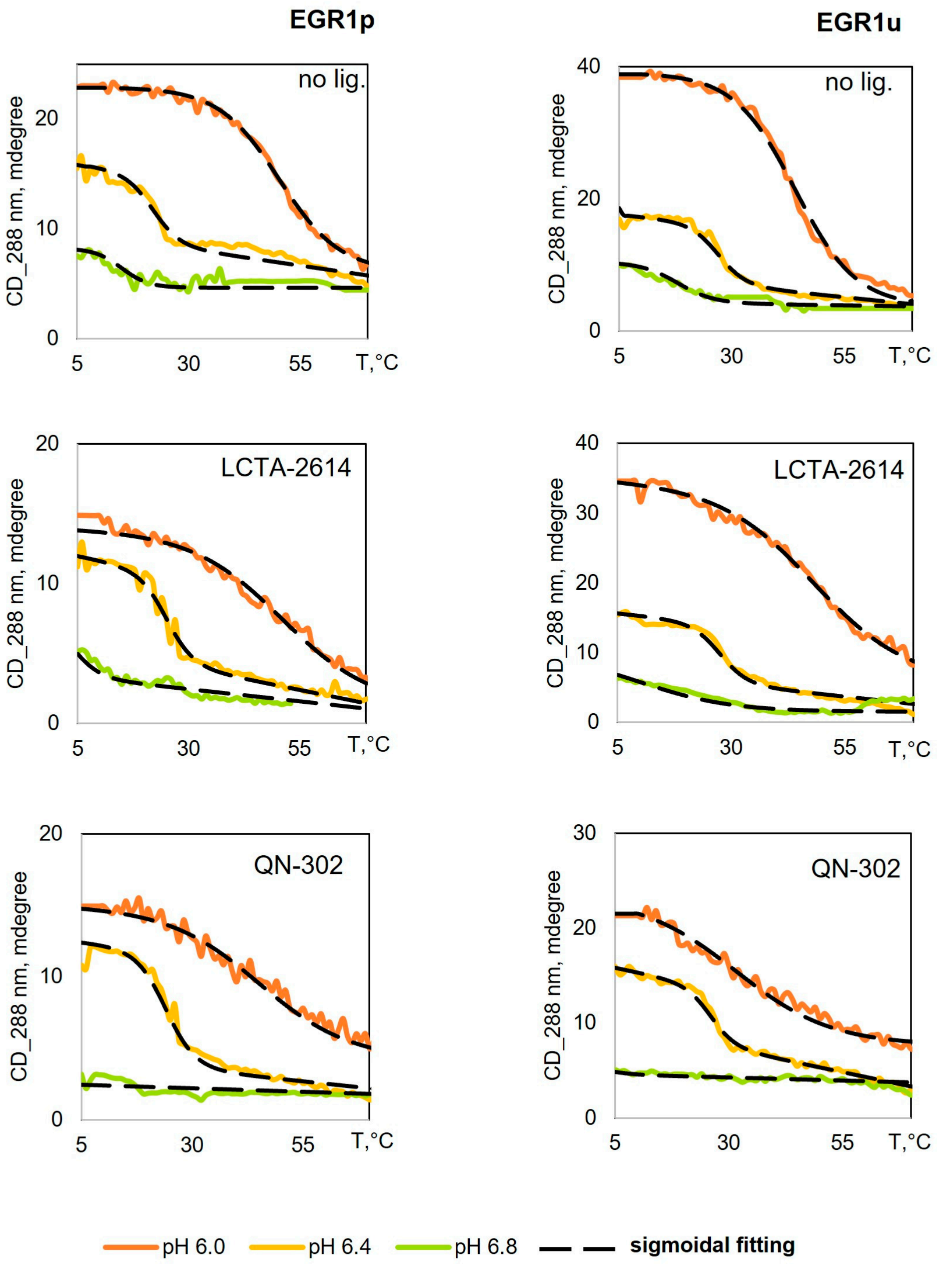

Figure S10. Melting curves of ERG1p and EGR1u iMs in the absence and in the presence of the ligands. Conditions: 3 µM iM and 20 µM LCTA-2614/QN-302 in 10 mM sodium phosphate buffer supplemented with 140 mM KCl and 20% PEG-400.

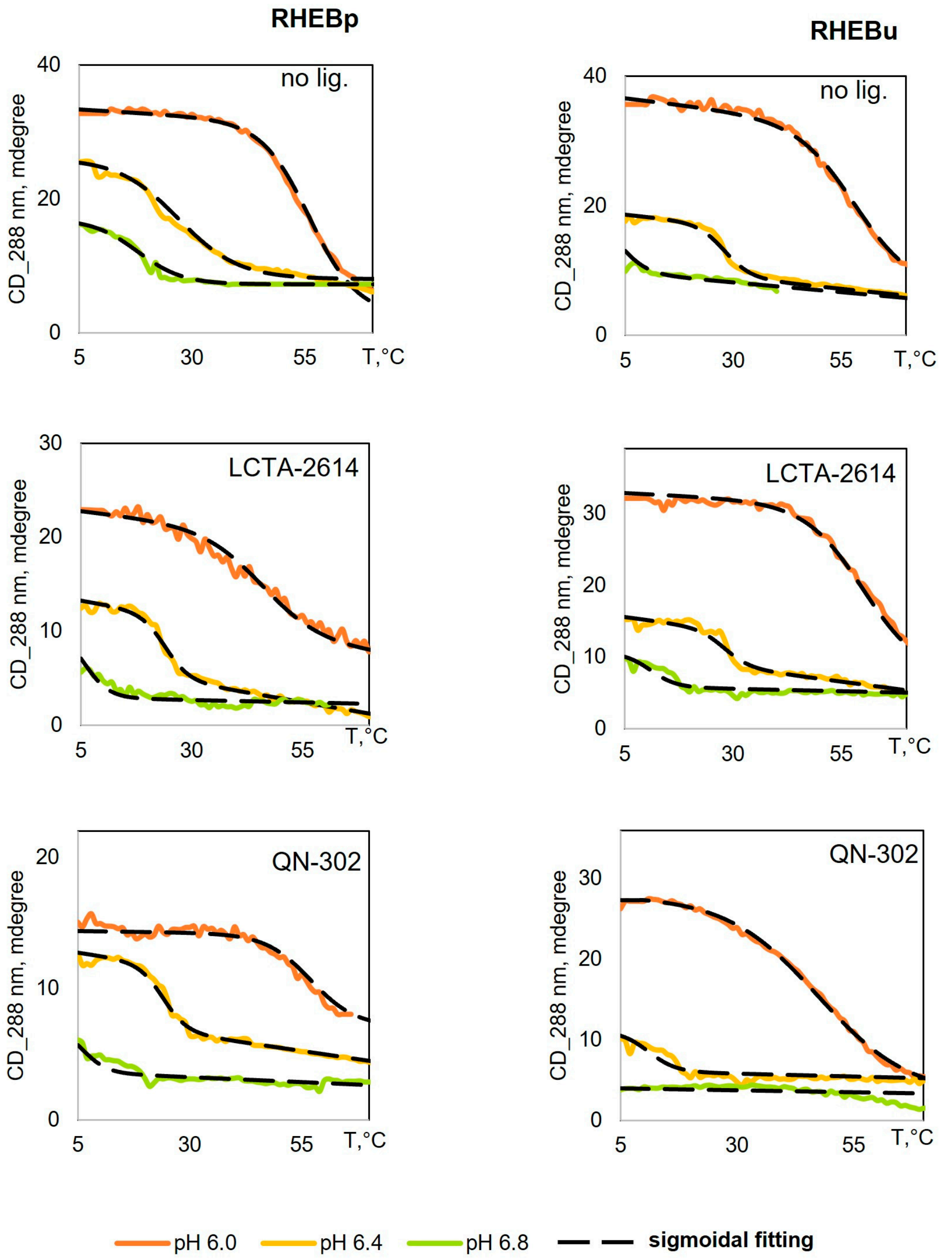

Figure S11. Melting curves of RHEBp and RHEBu iMs in the absence and in the presence of the ligands. Conditions: 3 μM iM and 20 μM LCTA-2614/QN-302 in 10 mM sodium phosphate buffer supplemented with 140 mM KCl and 20% PEG-400.
